# FEASIBILITY OF AN EXERGAMING-BASED DANCE TRAINING PARADIGM IN THE HOME SETTING AMONG PEOPLE WITH CHRONIC STROKE

**DOI:** 10.1093/geroni/igac059.2385

**Published:** 2022-12-20

**Authors:** Savitha Subramaniam, Tanvi Bhatt

**Affiliations:** University of Illinois at Chicago, Chicago,, Illinois, United States; University of Illinois at Chicago, Chicago,, Illinois, United States

## Abstract

**Background:**

Dance-based exergaming (DBExG) from laboratory (phase 1 -p1) to a safe and feasible home-based exercise program (HEP- phase 2-p2) could enhance physical activity (PA) behavior, and can be used as a maintenance therapy in PwCS. However, traditionally structured HEP are interfaced with impediments for employing best practice for fall prevention safety approach at home settings in PwCS, thus, making it essential to evaluate training strategies that foster safety. Aim: To evaluate the feasibility, compliance, and safety of a safety harness augmented rehabilitation using DBExG training paradigm (SHARP).

**Methods:**

Community-dwelling PwCS (n=7) participated in the study and received DBExG training using the commercially available Kinect dance gaming “Just Dance 3”. The first 6 weeks training (20 sessions) was provided in the laboratory setting (health coach stand by assistance – SBA). Followed with 4 more weeks (12 sessions) of SHARP in the participant’s house with health coach SBA.

**Results:**

The primary focus was feasibility, addressed by acceptability, and retention. All the seven participants completed the laboratory, and home-based DBExG. All participants reported enjoying the sessions and felt they were beneficial. Study retention and session adherence was 90% and 98%, at p1, and p2 respectively. There were no falls, and adverse safety events reported in either phase of the study. The intervention was safe with no falls, and major adverse events.

**Conclusion:**

SHARP appears feasible and safe, thus promising for home-based PA rehabilitation for PwCS. A larger randomized controlled trial is recommended to further investigate efficacy.

